# Correlational study of impacted and non-functional lower third molar position with occurrence of pathologies

**DOI:** 10.1186/s40510-016-0139-8

**Published:** 2016-09-05

**Authors:** Igor Batista Camargo, João Batista Sobrinho, Emanuel Sávio de Souza Andrade, Joseph E. Van Sickels

**Affiliations:** 1 Department of Oral and Maxillofacial Surgery, Oral and Maxillofacial Surgeon of Brazilian Army, Rio de Janeiro, Brazil; 2 Department of Oral and Maxillofacial Radiology, Dental School at Pernambuco, UPE, Brazil; 3 Department of Oral and Maxillofacial Pathology, Dental School at Pernambuco, UPE, Brazil; 4 Department of Oral and Maxillofacial Surgery, University of Kentucky, Lexington, Kentucky USA

## Abstract

**Background:**

Lower third molar (M3) eruption is unpredictable. The purpose of this study was to correlate radiographic position of M3 on a preexistent film with the current clinical, histopathological, and radiographic findings.

**Methods:**

A retrospective cohort study was performed. The sample was collected from a database of patients covered by Medical Fund of Brazilian Army. Radiographs were obtained a minimum of 5 years prior to the presurgical visit and after their clinical exam. The primary outcome variables were the teeth positions using Pell and Gregory/Winter classifications on panoramic X-rays. Those variables were analyzed at both the beginning (T0) and end of the study (T1). Clinical assessments and histopathological study of the thirds that were extracted were performed only at T1. Correlation between the teeth positions were related to the clinical, histopathological, and radiographic parameters using statistical analysis tests with significance set at *p* < 0.05.

**Results:**

Twenty-six patients with 49 M3 were assessed over 10 months. Mean age was 14.92 years at T0 and 21.87 years at T1. The average time between T0 and T1 was 6.77 years. A significant relationship (*p* = 0.024) was found between the presences of root resorption on the second molar if M3 presented in an IB horizontal position at T1. There was also a significant correlation (*p* = 0.039) between dental crowding of the anterior lower teeth with IIIB position at T0 and if the patient finished orthodontic treatment without lingual retainers.

**Conclusions:**

Lower M3 in position IIIB seen in a teenager and IB seen in an adult is more likely to have negative consequences and should be followed closely.

## Background

The third molar (M3) eruption is an unpredictable event. The average age for eruption is 20 years, with a range from 14 to 24 years [1–3]. It is generally accepted that racial variation in facial growth, jaw size, and tooth size is crucial to the eruption pattern and impaction status. Longitudinal studies on positional changes and eruption have been conducted in Western populations [4–6]. Studies from other populations indicate that some non-erupted M3s reach the occlusal plane in the third decade of life [5–7]. No such data are available for South America patients. Regardless of ethnic background, third molars have the highest rate of impaction of all teeth [8–10]. A lack of space in the arches is a common cause of impaction of the third molars [11–16]. When remodeling/resorption in the anterior region of the mandibular ramus is limited, M3s become impacted [12–14, 16].

In observation studies, the relationship between the third and second molars over time can lead to the development of pathologic conditions such as caries, pericoronitis, dental crowding, second-molar root resorption, and odontogenic cysts [17–22]. As such, many mandibular M3s are removed prophylactically prior to the onset of significant problems [23–35].

However, third molars may be beneficial for orthodontic and restorative treatment. A number of authors have shown that they become upright throughout development as their angulation improve [17, 26–28]. Additionally, mesial migration of the molars due to interproximal attrition or extraction therapy increases the eruption space and reduces the frequency of the third molar impaction [18, 19, 29, 30].

Knowledge of the fate of asymptomatic M3s after early adulthood is required in order to make an informed decision regarding their removal. The purpose of this study was to correlate radiographic position of the third molars on preexistent films with the current clinical, histopathological, and radiographic findings. The investigators hypothesize that the third molars that developed in a deep impaction pattern according to Pell and Gregory and Winter classifications can be associated with local changes and pathologies. Hence, the aim of this study is to determine angular changes and the eruption level of impacted third molars in Brazilian subjects over a 5-year observation period (longitudinal retrospective cohort) in a group of patients referred for surgical evaluation of the third molars. The specific aims were to evaluate the angular and positional clinical changes in the mandibular third molars over time and correlate with the development of pathology.

## Methods

To address the research purpose, the investigators designed and implemented a cohort study in which the sample was collected between January and October 2008. The study population was composed of patients assisted and treated by the “Fundo de Assistência Médica do Exército” (Medical Assistance Fund of the Brazilian Army—SAMMED/FUSEX) at the Recife Military Area Hospital (HMAR) from the Odontological Clinic. The subjects selected for the study were collected from a database of panoramic radiographs from patients who were initially referred between 1997 and October 2003 to a private radiological clinic—Radioface—in Recife, Pernambuco (Brazil).

### Data sampling

Patients of the study population were previously referred for removal of impacted and non-functional third molars or were contacted by phone based on the data banking cited above (T1). The patients had been seen in the same facility clinic in HMAR, with a minimum of 5 years of care and had a panoramic radiograph taken (T0). Inclusion criteria for the study were the following: (a) patients presenting for treatment between 1997 and 2003, who were between 9 and 27 years old; (b) incomplete root formation (sixth stage of Nolla at T0); (c) root formation with at least two thirds of the complete root at T1 (Nolla stage 8); (d) intact lower posterior arch with at least all first and second molars and two premolars one at the side of the M3; and (e) excluding criteria was patients older than 30 years at T0 and those with incomplete records.

All subjects of research had a panoramic radiograph prior to start orthodontic or general dental treatment (T0). At a minimum of 5 years later (T1), patients were examined both clinically and radiographically. After the patients were informed of the diagnosis and had indication and consented to treatment, they had their teeth extracted. The pericoronal tissue (of all tooth extracted) was then sent for histological examination at the pathology laboratory of the “Faculdade de Odontologia de Pernambuco” (College of Dentistry of Pernambuco State—FOP/UPE). The study was approved by the Research Ethics Committee of the UPE (project number: 194/07).

The predictor variable was time (T0 and T1). The primary outcome variable was teeth position. It was analyzed at both the beginning (T0) and end (T1) of the study. The secondary outcome variables were the following: the clinical findings; root formation; width of pericoronal radiolucency; bone coverage; and histopathological analysis of the collected follicles. Other variables studied were age and sex. The assessments of clinical and histopathological outcome variables were performed only at the end (T1) as described in the following sections.

### Clinical examination

Physical examination of the oral cavity was performed to confirm the presence or absence of the third molars. Based on the clinical examination, the thirds were classified as erupted, partial erupted, and full bony impactions. The presence or absence of dental caries in the third and second molars was noted. White patches on either or the teeth were not considered dental caries. Pericoronitis and periodontal disease was also recorded by periodontal probing and by visualization of purulence in the region of the third molar. Finally, the presence or absence of the expansion of cortical bone surrounding the third molar and the presence or absence of crowding of the first and second molars was noted. Patients were asked whether they had orthodontic treatment and, if so, the presence or absence of orthodontic retainer in the anterior mandibular teeth was recorded. Mucosal coverage at T1 was the only parameter that was assessed from the patient’s histories. The teeth were divided into M3s without mucosal coverage, partial mucosal coverage, and those with total mucosal coverage.

### Radiographic evaluation—the third molar classification

**Fig. 1:**
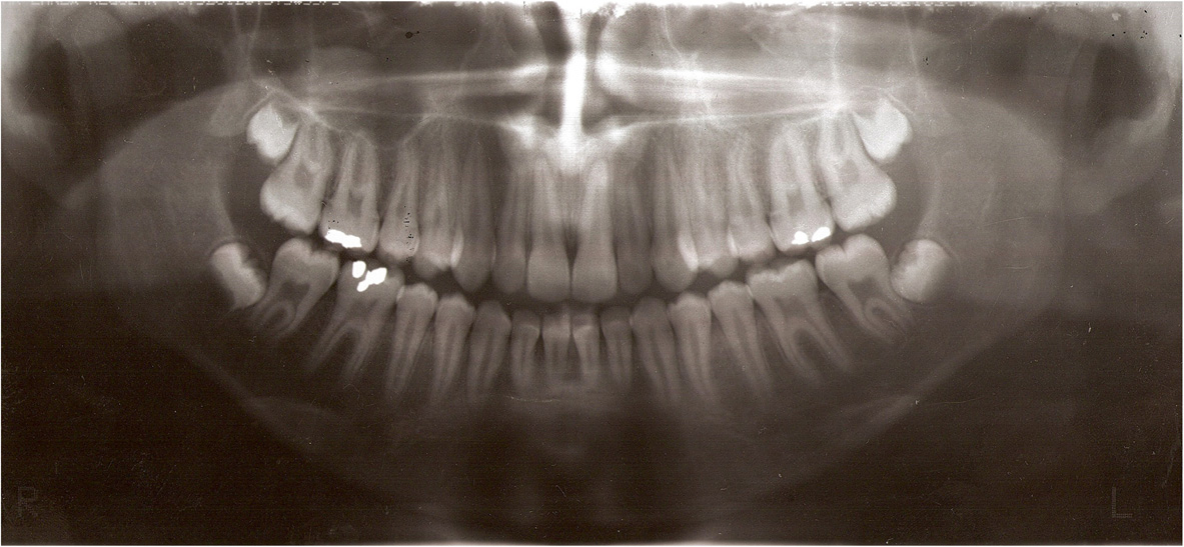
Panoramic radiograph at T0

**Fig. 2:**
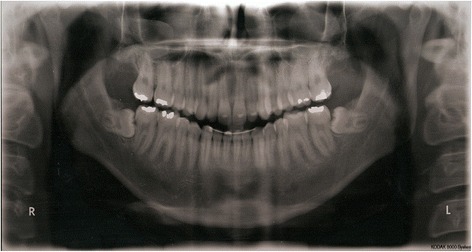
Panoramic radiograph of the same patient showing the M3 development along 5 years (T1)

**Fig. 3:**
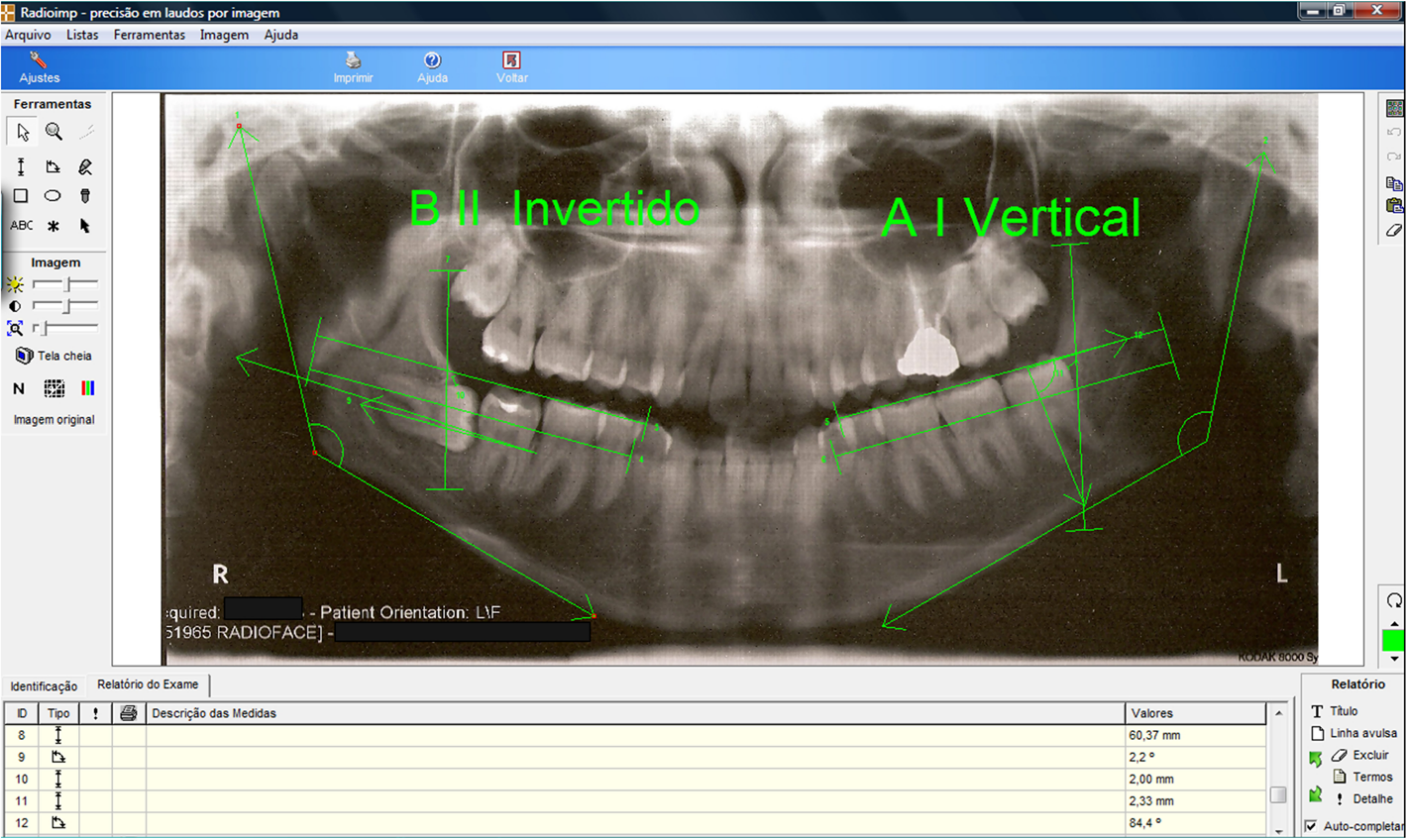
Radioimp® software used to obtain the M3 classification and dental follicle measurements

All subitems listed below were analyzed with paired *t* tests between groups and individual third molar (patients at T0 (1997–2003) (Fig. 1) and at the end of the study—T1 (2008) (Fig. 2). The position of the mandibular M3s was assessed using a series of parameters. The teeth were distributed in four different subclasses based on position variables using the Pell and Gregory classification, the Winter classification, the degree of mucosal coverage, and the degree of bone coverage [31]. Tooth position parameters were radiologically assessed using the Radio Memory software program (Radio Memory Ltd., Version 3, Release 3.1, Belo Horizonte, Brazil) and by tracing four lines on previously scanned orthopanoramic radiographs (resolution 350 dpi). These lines were established to provide (1) the line of the occlusal plane, established by the occlusal surfaces of the lower first and second molars; (2) the cervical line, delineated by the cervical bone limit of the lower second molar; (3) the line of the lower margin of the ascending mandibular ramus; and (4) the longitudinal axis of the M3. From these lines and measurements, the M3 was classified according to depth (Pell and Gregory positions A, B, and C) and the degree of M3 impaction with respect to the ascending ramus (Pell and Gregory classes I, II, and III) as well as the absence or presence of partial or total bone coverage. The Winter classification was obtained using the fourth line that formed an angle between the M3 and the occlusal plane. It allowed to have the objective subclasses as follows: (1) M3s with negative angles (<0°) were considered inverted; (2) M3s with an angle between 0° and 30° were considered horizontal; (3) M3s with an angle between 31° and 60° were considered to be mesioangular; (4) M3s with an angle between 61° and 90° were considered vertical; and (5) M3s with an angle >90° were considered distoangular (Fig. 3). Likewise, M3s were classified based on bone coverage into three subtypes: no bone coverage, those with partial bone coverage, and those with complete bone coverage. With this, the teeth were classified as to the stage of eruption of the tooth as erupted, partial erupted, or full bony impacted.

### Radiographic evaluation—root formation and width of pericoronal radiolucency

The formation of M3 roots was classified based on the Nolla stage of root formation. The width of pericoronal radiolucency was measured electronically in millimeters as the greatest width from the middle of the mesial, distal, and occlusal planes either alone or simultaneously to the edge of the bone (Fig. 3).

### Statistical analysis

Changes in M3 angulation and eruption level were tabulated separately for mandibular M3s at the baseline and at the 5-year minimal follow-up. Root formation was also evaluated over time (T0 and T1 radiographs). Statistical analysis of the data was performed using the paired Student’s *t* test, independent Student’s *t* test, analysis of variance, and the chi-square (*χ* ^2^) test. Tooth position variables were statistically related to the clinical parameters using SPSS version 13.0 statistical package for Microsoft Windows (SPSS Inc., Chicago, IL). The following analyses were performed: correlation matrix among all the study variables; global or symmetrical measures of association (McNemar test for categorical data, Pearson’s chi-square, and Fisher’s exact test); directional measures of association (*F* test—ANOVA and Tukey’s comparisons or Tamhane’s comparisons). The margin of error to assess significance for all statistical tests was 5.0 %.

## Results

In total, 74 patients met the initial inclusion criteria and who still had their third molars and were invited to participate in this study (T1) (Fig. 1). Of these, 26 patients with a total of 49 impacted or non-functional third molars agreed to compose the study. Of the 74 patients who were initially contacted to participate in the study, 64.9 % chose not to participate. In some cases, it was secondary to their military assignments. Some had been transferred to distant location and chose not to travel to the city of Recife (6.7 %). Others chose not to participate after the study was described to them (48.6 %). And finally, after the clinical examination at T1, the rest did not meet the inclusion criteria.

Twenty-six patients were recruited; the average follow-up of this cohort study was 6.77 years. Seven (26.9 %) were male and 19 (73.1 %) were female. At T0, they were between 9 and 26 years (median, 14.9 years; standard deviation ± 4.13 years; coefficient of variation, 27.3 %). At T1, they were between 14 and 32 (median, 21.9 years; standard deviation ± 4.45 years; coefficient of variation, 20.3 %). In total, 49 mandibular M3s were assessed. The observation time ranged from 5.04 to 10.2 years; there was a mean of 6.77 ± 1.50 years. The median value was 6.55 years.

Table 1 presents the clinical finding result data which highlights the following: slightly over half (51.9 %) of the third molars were classified as full bony impacted at T1, followed by 26.9 % as partial erupted and 15.4 % were erupted. The majority (81.6 %) had no caries in the third molars. Of those who had cavities in these teeth, the highest percentage (16.3 %) corresponded to those that had pits and fissures. Slightly over half (53.1 %) of the second molars showed no decay, followed by 42.9 % that had restorations in the occlusal plane. There were three of the third molars where pericoronitis was noted. None of the other third molars in this sample had periodontal disease or expansion of the surrounding cortical bone.

**Table 1:** Classification and prevalence of dental diseases analyzed according to the clinical data (T1)

Variable	*n*	Percent
• Stage of eruption—3rd molars		
Erupted	8	15.4
Partial erupted	14	26.9
Full bony impacted	27	51.9
Agenesis	2	3.8
Extracted as a result of pain	1	1.9
Total	52	100.0
• Cavity—3rd molars		
Absent	40	81.6
Pits and fissures	8	16.3
Cavitation	1	2.0
• Cavity—2nd molars		
Absent	26	53.1
Pits and fissures	2	4.1
Restored	21	42.9
• Pericoronitis—3rd molars		
Absent	46	93.9
Present	–	–
Previous episode at the site	3	6.1
• Periodontal disease—3rd molars		
Present	49	100.0
Absent	–	–
• Expansion of the cortical bone—3rd molars		
Absent	49	100.0
Present	–	–
Total	49	100.0

*n* number of teeth

Most (69.2 %) patients did not have crowding, and of those that did, 11.5 % involved the anterior teeth, 15.4 % involved the posterior teeth, and one patient had both. Only one patient had not undergone orthodontic treatment. Of those that did, most had completed treatment without anterior retainers (34.6 %), followed by 30.8 % with anterior retainers, while 23.1 % were in the midst of active orthodontics.

In the baseline evaluation (T0), the position of the teeth, according to the Winter classification, 67.3 % were classified as mesioangular. While at T1, the highest proportion were classified as vertical (40.8 %), followed by 26.5 % that were classified as mesioangular and 18.4 % as horizontal.

With the exception of one case where the M3 that was full erupted at T0, all the others had bone/soft tissue coverage. The majority (63.3 %) were completely covered with bone. At the final radiographic evaluation (T1), 57.1 % had partial coverage and the remaining 42.9 % had no coverage visualized on the panoramic images.

The width of radiolucency ranged from 0.00 to 3.39 (median, 1.74; standard deviation, 0.83). Of the 49 teeth evaluated, the presence of resorption of the distal root of the second molar was noted in 10 (20.4 %) cases and was absent in 39 ones (79.6 %). Resorption was identified by the presence of radiolucency in the distal root of the second molar.

Table 2 shows that the mean difference (variation) of the angle of inclination of the tooth increased by 7.2° from T1-T0. Additionally, there was an increase in the angle of 3MI teeth over the time. There was a natural tendency to become more vertical and erupt, even with a lack of space.

**Table 2:** Statistical analysis of the steepness changing of the third molars during the evaluation period

		Time of evaluation			
Variable	Statistics	Initial (T0)	Final (T1)	Difference	*p* value
• Angle	Average	49.43	56.63	7.20	*p* = 0.079^a^
	Median	53.80	58.90	9.20	
	SD	16.52	33.77	28.11	
	CV	33.42	59.63		
	Minimum	−3.80	−39.60	−48.80	
	Maximum	91.70	111.40	65.10	

*SD* standard deviation, *CV* coefficient of variation, *n* number of teeth

^a^Through the paired Student’s *t* test

Histological examinations were conducted in the 19 third molars that had to be extracted for orthodontic reasons. The dental follicle was identified in 15 (78.9 %). A dentigerous cyst was identified in four cases (21.1 %).

### Clinical-radiographic correlations

#### Influence of 3MI positioning at the baseline and end of the study on root resorption on mandibular second molars

The prevalence of root resorption of the second molars was evaluated with each of several variables (Table 3): stage of eruption as well as Pell and Gregory and Winter classifications at T0 and T1. Based on these data, the following trend was noted: when 3MI were at the end of its eruptive process (T1) and present in the IB/horizontal full bony impacted position, they have a higher probability of causing root resorption of the second lower molars (*p* = 0.024/*p* < 0.001).

**Table 3:** Correlation of the root resorption of second molars according to the stage of eruption of the lower M3 positions at T1

	Resorption—2nd molars						
Variable	Present		Absent		Total		*p* value
	*n*	%	*n*	%	*n*	%	
• Stage of eruption							
Erupted	–	–	8	100.0	8	100.0	*p* ^a^ = 0.005*
Partial erupted	–	–	14	100.0	14	100.0	
Full bone impacted	10	37.0	17	63.0	27	100.0	
Total	10	20.4	39	79.6	49	100.0	
• Pell and Gregory—T1							
IA	–	–	17	100.0	17	100.0	*p* ^a^ = 0.024*
IIA	3	30.0	7	70.0	10	100.0	
IB	3	37.5	5	62.5	8	100.0	
IIB	1	11.1	8	88.9	9	100.0	
• Winter—T1							
Horizontal	5	55.6	4	44.4	9	100.0	*p* ^a^ < 0.001*
Mesioangular	5	38.5	8	61.5	13	100.0	
Vertical	–	–	20	100.0	20	100.0	
Group total	10	20.4	39	79.6	49	100.0	

Obs.: Relative risk was not determined due to the occurrence of zero or very low frequencies

*n* number of teeth

*Significant difference at 5 %

^a^Fisher’s exact test

#### Influence of orthodontic treatment and dental positioning of 3MI related to dental crowding

Table 4 shows that the variable tooth position according to the Pell and Gregory classification at T0 had orthodontic treatment which were associated with the occurrence of dental crowding at T1. As it can be expected, dental crowding was zero among the patients who were still under the orthodontic treatment. Dental crowding was seen in only one case among the eight who had completed treatment and had anterior retainer and in one of two patients who stopped orthodontic treatment before it was competed. It could be concluded that the population with teeth in the IIIB position at the beginning of their root formation (T0) that undergo orthodontic treatment and finish that without use retainers have a larger chance of developing dental crowding in the anterior teeth (*p* = 0.039).

**Table 4:** Evaluation of the prevalence of crowding presenting with orthodontic treatment (T1) and the initial Pell and Gregory classification (T0)

	Dental crowding						
Variable	Present		Absent		Total		*p* value
	*n*	%	*N*	%	*n*	%	
• Orthodontic treatment—T1							
In implementation	–	–	6	100.0	6	100.0	*p* ^a^ = 0.039*
Completed without lingual retainer	5	55.6	4	44.4	9	100.0	
Completed with contention	1	12.5	7	87.5	8	100.0	
Not completed	1	50.0	1	50.0	2	100.0	
Not taken	1	100.0	–	–	1	100.0	
• Pell and Gregory—T0							
IIB	3	21.4	11	78.6	14	100.0	*p* ^a^ = 0.039*
IIIB	4	66.7	2	33.3	6	100.0	
IC	–	–	6	100.0	6	100.0	
IIC	7	50.0	7	50.0	14	100.0	

Obs.: Relative risk was not determined due to the occurrence of zero or very low frequencies

*n* Number of teeth

*Significant difference at 5 %

^a^Fisher’s exact test

#### Relationship between 3MI positions with pericoronal radiolucency width

The average radiolucency width for the final horizontal and vertical evaluation using both Pell and Gregory and Winter classifications are shown in Table 5. The results confirm that there was a relationship between the tooth and the occlusal plane (position B), which increased the probability of extending the pericoronal area. So, at T1, the tooth that has developed and acquired a position vertical and B (impacted) tends to show greater probability to have a pericoronal radiolucency width wider when compared to the vertical and A (semi-impacted or erupted).

**Table 5:** Statistic analysis of the width of radiolucency according to the Pell and Gregory and final Winter’s classification at T1

	Width of radiolucency						
Variable	Average	Median	SD	CV	Minimum	Maximum	*p* value
• Pell & Gregory—vertical at T1							
A	1.35	1.41	0.89	65.93	0.00	3.13	*p* ^a^ = 0.014*
B	1.98	1.83	0.61	30.81	0.93	3.39	

Obs.: *p* = 0.014*a

*Significant difference at 5 %

^a^Through the Student’s *t* test with equal variances

## Discussion

Third molars are the last teeth to erupt; due to this late eruption, they account for 98 % of all impacted teeth. Considering the numerous complications that can occur with their presence, the assessment of tooth position and progress of M3 eruption is necessary to optimize patient management [8–10].

In recent years, a number of authors have noted a relationship between the types of complication to anatomical parameters associated with the impacted third molar [32–34]. Kay [32] reports a close relationship between the mesioangular position and the appearance of pericoronitis. Wallace [33] attributes nearly 90 % of such cases to M3s in the vertical position. More recently, Leone et al. [34] have argued that M3s in the vertical position or slightly distoangular with partial mucosal and bone coverage are the presentations most likely to cause pericoronitis. Alcaraz et al. [35] found M3s in positions IB and IIB of the Pell and Gregory classification to be more closely associated with the appearance of pericoronal infection. In contrast to these reports, we did not find positive correlation between the tooth position and these types of complications (Table 1).

Follow-up studies of young adults noted radiographic changes in the sagittal projection continue to occur throughout the development of M3s in the pre-eruptive period. Three types of movements are observed in the sagittal projection: the M3 either becomes more upright, becomes more medially inclined, or remains unchanged [6]. During the observation period in the present study, 100 % of the teeth changed their sagittal inclination, 67.3 % exhibited uprighting changes (a decrease in angulation), and 24.5 % became more deeply inclined in the mandible (Table 2).

One hundred percent of the distoangular teeth in the mandible erupted to the occlusal level during the follow-up period in the present study. Kruger et al. [36] reported the eruption of less than one third (31.6 %) of mandibular distoangular teeth. In contrast, Hattab [3] suggests that mandibular impacted mesioangular M3s are unlikely to erupt in the third decade of life. In the present sample, 20 of 33 (60.3 %) mandibular mesioangular teeth became vertical during follow-up. Only 28 of the 49 mandibular teeth (57.1 %) erupted to the occlusal level, of which 13 teeth (26.5 %) remained mesioangular and 20 teeth (40.8 %) became vertical. Of these 20 vertical teeth, one tooth had an initial inclination of 25° or more and another tooth had an initial inclination of 35° or more. In a study involving subjects with a median age 25.9 years and a follow-up period of 2.2 years, Nance et al. [37] found that 11 % of the teeth angled 25° or more and 3 % of teeth angled 35° or more erupted to the occlusal plane [6]. Hattab [3] studied 59 mesioangular mandibular M3s in 36 patients (mean age at baseline 19.7 years) and found that 25 teeth (42 %) erupted into occlusion during the 4-year observation period. Only 3 % of teeth angled 25° or more and none angled 35° or more erupted to the occlusal plane, which is comparable to the findings of the present study. In our results, more than one third (34.7 %) of the impacted vertical teeth (levels B and C) in the mandible erupted to the occlusal plane (position IA) during the 5-year minimal follow-up period. Similar results have been reported in a study [37] involving 237 young adults (aged 14 to 45 years at enrollment, median age at baseline of 25.9 years), in which nearly one third of impacted vertical/distal M3s in both jaws erupted to the occlusal plane during a follow-up period of 2.2 years (29 % in the mandible). However, in a study involving subjects with a mean age of 18 years at the baseline and an 8-year follow-up period, Kruger et al. [36] reported the eruption of more vertically impacted teeth in the mandible (49.5 %) in comparison to the findings of the present study.

Root resorption on the second molars is one of the complications associated with the third molar impaction and most of the available data have come from case reports [38–40]. Knutsson et al. [41] evaluated 666 patients before removal of mandibular third molars and found that 548 patients had pathologic conditions (occlusal and/or distal caries, second molar root resorption and pericoronaritis). Root resorption of the second molar was found on the radiographs in 1 % of these cases. The prevalence of root resorption has been low in most studies, ranging from less than 0.3 to 7 % [39, 42]. Nemcovsky et al. [43] found a greater prevalence of root resorption (24.2 %) in a sample of 186 periapical radiographs of the third and second molars, similar to the value in the present study on the panoramic images (20.41 %).

The published data have shown that the position and angulation of third molars can have an effect on the pathologic features and symptoms associated with impacted third molars [44, 45]. Oenning et al. [46] studied the position using cone beam computed tomography (CBCT) and found that mesioangular and horizontal inclination was related to resorption of the second molar roots. There was a greater prevalence of root resorption with mesioangular than with distoangular position of the thirds. In the present study, IB/horizontal full bony impacted position third molars were statistically associated (*p* = 0.024/*p* < 0.001) with root resorption of second molars. These findings have corroborated studies that evaluated this relationship using conventional radiographs [43]. The mesial inclinations, mesioangular and horizontal, have a larger area of contact between the third and second molars, leading us to believe that the inflammatory process will be more severe and has a greater potential to damage the dental surface.

As the evidence supporting surgical therapy versus surveillance for disease-free third molars is lacking [47], we believe that the position of the tooth to the adjacent second molar should be considered during the decision-making process [43]. When root resorption is suspected, CBCT may help when considering extraction of the third molar. Although resorption of the second molar roots is thought to be rare, assessing this potential outcome should be included in a monitoring protocol.

Develop longitudinal cohort studies with large samples have difficulties related to the patient compliance and follow-up issues. In our study, we had a significant sample lost between the patients that matched our initial inclusion criteria (48 of 74). For this reason, it is important to reinforce that we found that the position IIIB at beginning of the development of the lower third molar was associated with dental crowding in patients without retainer in the end of their treatment. However, we could not statistically correlate this result with the age, sex, and orthodontic treatment because the sample size was not large enough for this proposal. Therefore, the dental crowding found in our sample could be related to the growth and mandibular development and not only by the third molar eruption process.

There is a controversy about the size of the pericoronal radiolucency surrounding an impacted third molar [7, 9, 48]. Stephens et al. [49] suggest the probability of a cyst with a pericoronal space of more than 2.5 mm. According to Glosser and Campbell [50], the radiographic appearance of a cyst was defined as a pericoronal radiolucency of 2.5 mm. In our study, the teeth that were vertical but in position B tended to have a wide pericoronary radiolucence surrounding them. Conklin and Stafne [51] reported a relationship between the width of the pericoronal space and the presence of an epithelial lining associated with an impacted tooth. Glosser and Campbell [50] conducted a histological examination of dental follicles associated with radiographically normal impacted M3s and found a higher incidence of dentigerous cystic changes in approximately 37 % of mandibular molars.

Due to the number of cystic changes that were found in the present study, careful histological examination is recommended when the teeth are extracted. In the present study, the incidence of cystic changes in impacted mandibular M3s was 21.1 %.

## Conclusions

The third molars with position IIB may have a greater risk of widening the pericoronal space. A correlation was found between the patients who had presented with dental crowding and had undergone orthodontic treatment without containment and maintained the mandibular third molar by the end of treatment. The prevalence of root resorption on the distal surface of mandibular second molars was found to be highly associated with horizontal angulation and position IB in the present study. Therefore, clinicians should consider lower M3 in position IIIB seen in a teenager and IB seen in an adult, once they are more likely to have negative consequences and should be followed closely. Further future studies following cohort methodology with large samples should be developed to better clarify those correlations.
